# MRI‐informed cortical features for brain age prediction in age‐specific adulthoods

**DOI:** 10.1002/hbm.26050

**Published:** 2022-08-16

**Authors:** Jing Li, Hanna Lu

**Affiliations:** ^1^ Department of Psychiatry The Chinese University of Hong Kong Hong Kong SAR China; ^2^ The Affiliated Brain Hospital of Guangzhou Medical University Guangzhou China

We read with great interest the study investigating the performance metrics of the “Brain‐age gap” prediction model in two world‐renowned large‐scale data sets (de Lange et al., 2022). Based on individual structural Magnetic Resonance Imaging (MRI) scans, the “Brain Age Gap Estimation” (BrainAGE) model can provide the quantitative estimation of morphometric changes during healthy and pathological aging (Silk & Wood, 2011). At present, the MRI‐informed BrainAGE score tends to be a promising neurophenotype to detect the individuals with accelerated brain atrophy or at high risk of developing age‐related neurodegenerative diseases (e.g., dementia) and further can serve as an indicator to differentiate the dementia patients from the healthy ones (Franke & Gaser, 2014). In the last decade, the brain age prediction building on Franke et al.'s pioneering study has extended to uncover the nonlinear brain atrophy in Alzheimer's disease (AD) using machine learning methods (Beheshti et al., 2020; Franke et al., 2010). Indeed, the selection of a robust machine learning model with high generalization ability in a general population is one of the major tasks before using BrainAGE in real‐world clinical practice. In de Lange et al.'s study, the BrainAGE score was calculated based on the structural MRI scans in adults aged over 45 (i.e., middle‐age to late adulthood). Considering the human brain is fully developed (i.e., maturity) around the age 25 (Foulkes & Blakemore, 2018), pre‐adulthood and young adulthood are also critical and valuable for calculating the BrainAGE score and in‐depth understanding the brain age changes in different stages of life (Figure 1).

**FIGURE 1:**
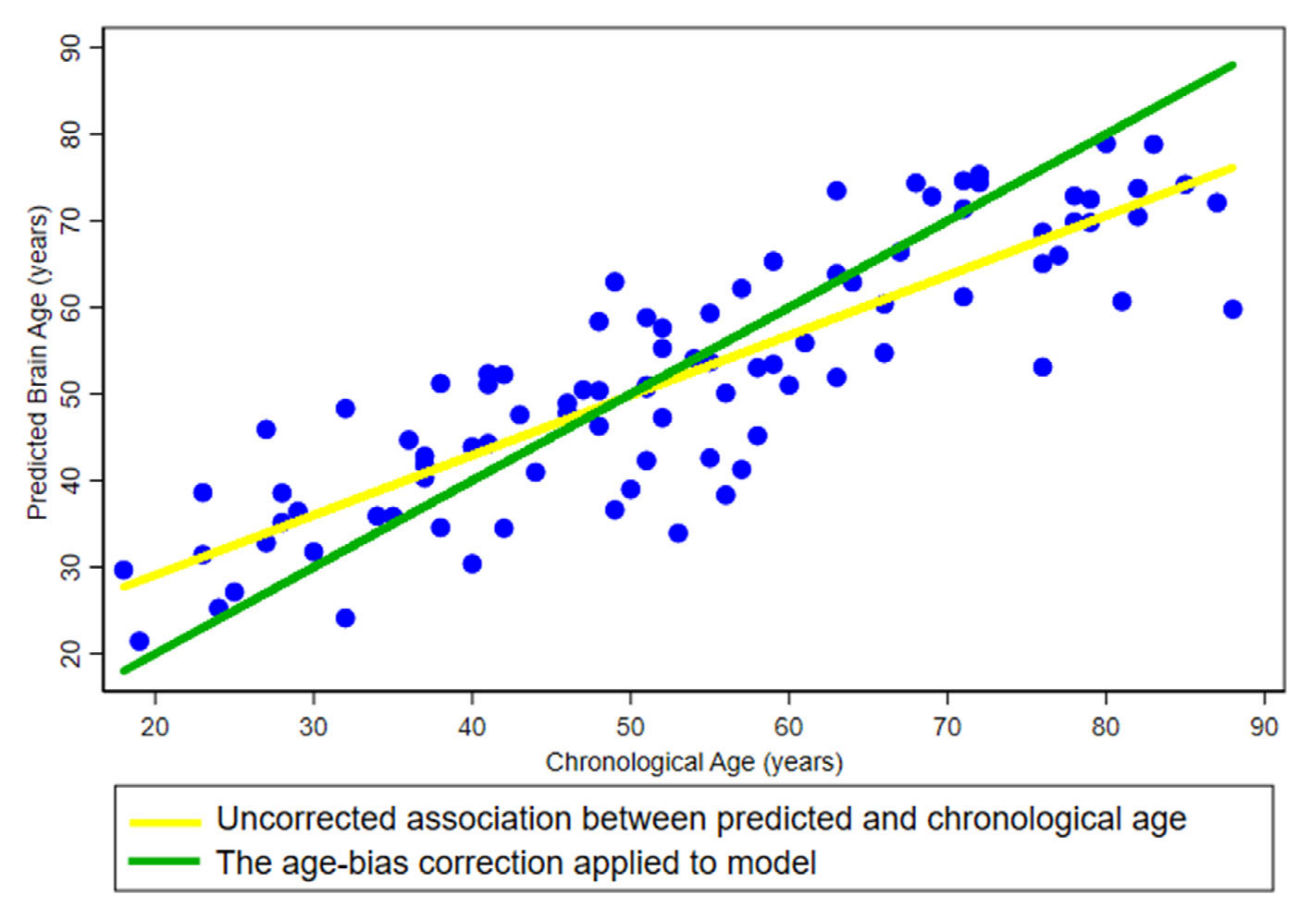
The illustration of MRI‐informed predicted brain age with or without age‐bias correction. The yellow line represents the uncorrected association between predicted brain age and chronological age. The green line represents the age‐bias corrected association between predicted brain age and chronological age.

Using the high‐resolution T1‐weighted structural MRI scans from the Cambridge Centre for Ageing and Neuroscience project (Cam‐CAN) project (*N* = 611; https://www.cam-can.org; Taylor et al., 2017), our team has recently investigated the performance metrics in individuals with different age ranges, including pre‐adulthood (aged from 18 to 22), young adulthood (aged from 23 to 40), middle adulthood (aged from 41 to 60), and late adulthood (aged from 60 to 90) using Support Vector Regression. The detailed steps of evaluating the performance metrics included: (1) based on the Cam‐CAN data, we randomly split the data set into two sets: 50% training set and 50% testing set. The age‐range in training set (*n* = 305) covered the whole adulthood (i.e., aged from 18 to 88). The testing set was further divided into three testing sets with same sample size (*n* = 71). (2) We calculated the *R* ^2^, mean absolute error (MAE) and root mean square error (RMSE) in three age‐specific adulthoods with the same age range (i.e., 18–38, 38–58, and 58–78 years) for the evaluation of performance metrics. An additional testing set (*n* = 71), including the whole adulthood (18–78 years) was also used for calculating the *R* ^2^, MAE and RMSE. (3) Age‐bias correction was applied to correct brain age prediction in the testing sets with the coefficients derived after fitting the training set.

As shown in Figure 2a, the patterns of *R* ^2^, MAE and RMSE changes were similar in the three testing sets with same age span (i.e., 18–38, 38–58, and 58–78 years) in the original models and the models with age‐bias correction. Greater value of *R* ^2^, representing a better model performance in testing sets, was accompanied with lower MAE/RMSE scores. Based on the *α* and *β* coefficients derived from the two models with or without age‐bias correction, we observed that the BrainAGE score showed the best prediction performance with the higher *R* ^2^ and lower MAE/RMSE scores in late adulthood (aged from 58 to 78 years) and whole adulthood (aged from 18 to 78 years).

Moreover, the discrepancies of sample size in training and testing sets were aligned with real‐world applications. For instance, the performance metrics of BrainAGE validated in the testing sets with a relatively small sample size (*n* = 71) shed light on the potential for the utilities to identify and quantify the individual brain age in clinical populations, such as AD and other types of dementia. Based on large‐scale imaging data sets, an accurate prediction model of BrainAGE can be successfully validated in a sample across full‐fledged adulthood. The method of age‐bias correction achieved an alignment between the predicted brain age and chronological age by changing individual's brain age estimation on the basis of the correction coefficients (i.e., *α* and *β*) derived from the training set (Beheshti et al., 2019). For example, after fitting the age‐bias correction coefficients from the training set, a child with a predicted brain age of 10 years gets an age bias‐corrected brain age of 4 years, and an elderly with a predicted brain age of 80 years gets an age‐bias corrected brain age of 94 years (Figure 1). In this regard, the age‐bias correction can only modify the model performance in the individuals within the age range same as training set. We observed improved values of *R* ^2^ in the testing sets with an age range of 38–58 (from −0.04 to 0.42) and the testing sets with an age range of 18–78 (from 0.77 to 0.86).

Meanwhile, the forced age‐bias correction could greatly offset even distort the actual degree of accelerated brain aging in senior adults or immature brain development in adolescents and young adulthood (Figure 2). We found decreased *R* ^2^ in the testing sets with an age range of 18–38 (from 0.16 to −0.35) and the testing sets with an age range of 58–78 (from 0.48 to −0.40). Interestingly, fitting the age‐bias correction with *α* and *β* coefficients derived from the corresponding testing sets can greatly improve the prediction accuracy in the testing sets covering the whole adulthood. The increased values of *R* ^2^ were found in the testing set with an age range of 18–38 (from 0.16 to 0.77), the testing set with an age range of 38–58 (from −0.04 to 0.77), the testing set with an age range of 58–78 (from 0.49 to 0.79) and the testing set with an age range of 18–78 (from 0.77 to 0.86) (Figure 2a).

**FIGURE 2:**
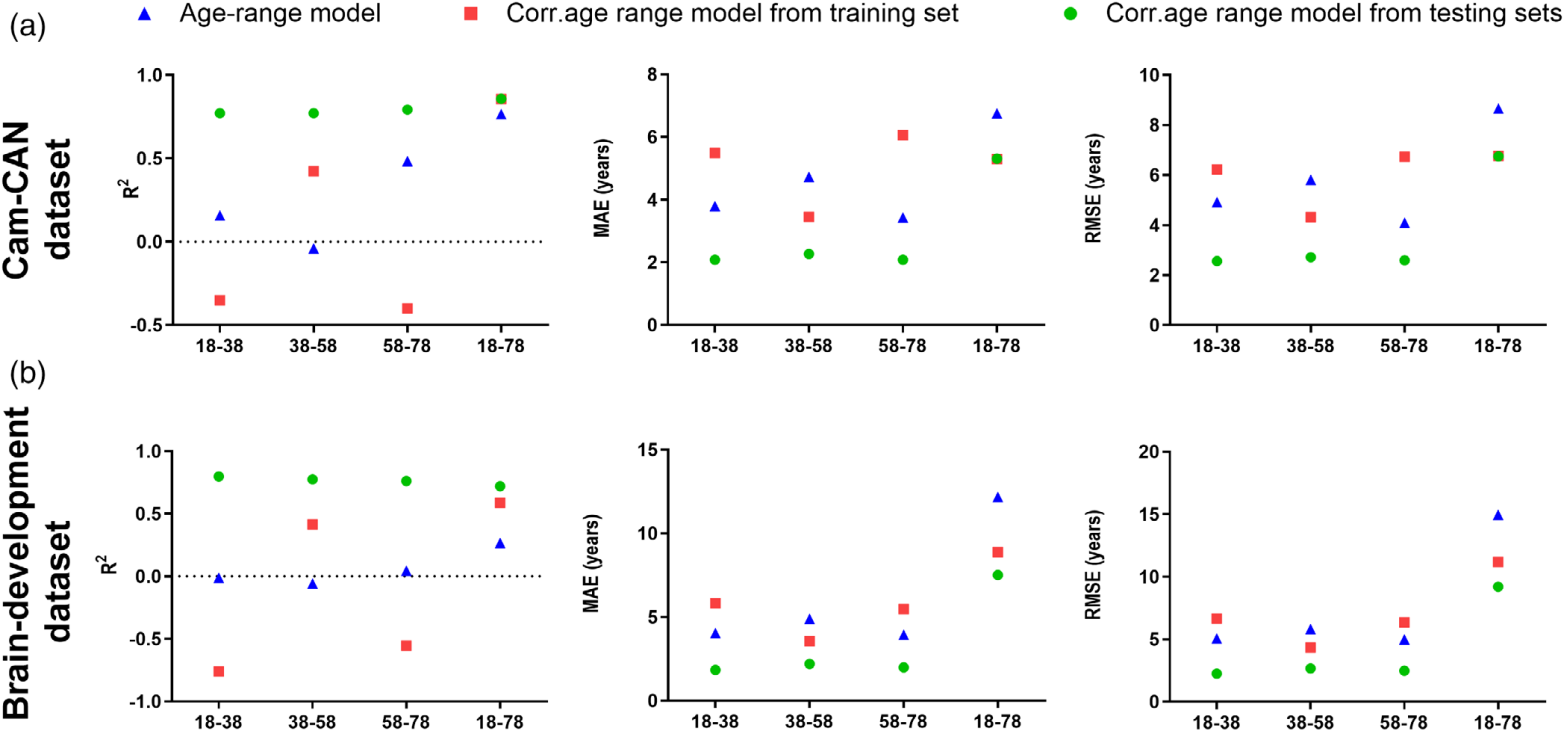
Performance matrices of MRI‐informed BrainAGE in age‐specific adulthoods. Performance matrices, including $R^{2}$, MAE, and RMSE, are calculated in the three models from the testing sets in cam‐CAN data set (a) and brain development data set (b). Blue triangle represents “The age‐range model” without age‐bias correction. Red rectangular represents “The Corr. Age‐range model from training set” with age‐bias correction derived *α* and *β* coefficients from training samples. Green circle represents “The Corr. Age‐range model from testing set” with the age‐bias correction derived *α* and *β* coefficients from the testing sets.

To further validate the results, we conducted the performance metric evaluation of BrainAGE in another data set from the Brain Development project (https://brain-development.org/ixi-dataset/), which includes structural MRI scans from 547 healthy participants with the age range of 19–86. We randomly selected four testing sets from the Brain Development data set that had the same age span (i.e., 18–38, 38–58, 58–78, and 18–78 years) and sample size (*n* = 71) as the Cam‐CAN data set had. Compared with the results from Cam‐CAN data set, similar patterns of BrainAGE changes were found in the individuals recruited from Brain Development data set (Figure 2b). After fitting the age‐bias correction coefficients derived from the training set, the decreased *R* ^2^ were observed in the testing sets with an age range of 18–38 (from −0.01 to −0.76) and the testing sets with an age range of 58–78 (from 0.05 to −0.55). Meanwhile, the increased values of *R* ^2^ were observed in the testing sets with an age range of 38–58 (from −0.06 to 0.78) in 38–58 years testing set and the testing sets with an age range of 18–78 (from 0.27 to 0.72). After age‐biased correction with the coefficients from a fit in the testing sets, the increased values of *R* ^2^ were found in the testing set with an age range of 18–38 (from −0.01 to 0.80), the testing set with an age range of 38–58 (from −0.06 to 0.78), the testing set with an age range of 58–78 (from 0.05 to 0.76) and the testing set with an age range of 18–78 (from 0.27 to 0.72; Figure 2b).

Taken together, as we look toward future research focused on brain age prediction, a healthy control group with same mean age and age range as the targeted clinical groups is necessary to generate the age‐bias correction coefficients when applying the BrainAGE as an aging biomarker in clinical populations.

In conclusion, we investigated the performance metrics of the brain age prediction models in individuals with different life stages. The trained model of brain age prediction has the best performance in late adulthood rather than other adulthoods. We further proposed a new method of age‐biased correction by fitting the correction coefficients derived from the control groups. This newly developed age‐biased correction method presents promising potential for future utilization of BrainAGE in age‐specific brain diseases.

## FUNDING INFORMATION

The Hong Kong Research Grant Council (RGC)‐General Research Fund (GRF), Grant/Award Number: 14111021)

## CONFLICT OF INTEREST

The author declares that there is no conflict of interest that could be perceived as prejudicing the impartiality of the research reported.

## Data Availability

Data sharing is not applicable to this article as no new data were created or analyzed in this study.
